# Repeated Induced-Membrane Technique Failure without Infection: A Series of Three Consecutive Procedures Performed for a Single Femur Defect

**DOI:** 10.1155/2020/8892226

**Published:** 2020-08-07

**Authors:** Laurent Mathieu, Marjorie Durand, Thomas Demoures, Christian Steenman, Alain-Charles Masquelet, Jean-Marc Collombet

**Affiliations:** ^1^Department of Orthopedic, Trauma and Reconstructive Surgery, Percy Military Hospital, 101 Avenue Henri Barbusse 92140 Clamart, France; ^2^French Military Health Service Academy, Ecole du Val-de-Grâce, 1 Place Alphonse Laveran 75005 Paris, France; ^3^Military Biomedical Research Institute (IRBA), 1 Place Général Valérie André, 91220 Brétigny-sur-Orge, France; ^4^Department of Orthopedic and Trauma Surgery, Bégin Military Hospital, 69 Avenue de Paris 94160 Saint-Mandé, France; ^5^Department of Orthopedic, Trauma and Hand Surgery, Saint-Antoine Hospital, 184 Rue du Faubourg Saint-Antoine 75012 Paris, France

## Abstract

A 40-year-old male was treated using the induced-membrane technique (IMT) for a noninfected, 9 cm long femoral bone defect complicating a lengthening procedure. The interesting case feature lies in the three consecutive IMT procedures that were necessary to achieve complete bone repair in this unusual clinical situation. The first procedure failed because of the lack of graft revascularization likely related to an induced-membrane (IM) alteration demonstrated by histological observations. The second IMT procedure led to partial graft integration interrupted by the elongation nail breakage. At last, the third procedure fully succeeded after nail exchange and iterative iliac bone grafting. Complete bone union was achieved with a poor functional recovery one year after the last procedure and four years following the first cement spacer implantation. By means of clinical and histological observations, we demonstrated that the first and the second IMT failures had two distinct origins, namely, biological and mechanical causes, respectively. Although simple, a successful IMT procedure is not so easy to complete.

## 1. Introduction

The induced-membrane (IM) technique (IMT) is a widely accepted method for the reconstruction of large segmental bone defects [1–7]. It is a two-stage procedure, involving a cement spacer in the first stage and a huge bone graft in the second stage. The spacer performs a mechanical action, given that it obviates fibrous tissue invasion of the recipient site, and a biological action via the induction of the surrounding membrane resulting from a foreign-body reaction [1–3]. Next, the membrane acts as a biological chamber to revascularize the bone graft and prevent it from resorption [3, 4]. However, mixed results have been reported, with specific concerns related to infective complications [5–7]. If complete definitive control of infection with appropriate soft-tissue coverage is a prerequisite to good bone union achievement, other factors may lead to IMT failure [2, 6, 7].

We report herein the unusual case of a patient with a noninfected femur bone gap complicating a lengthening procedure. Because of technical imperfections, the IMT was repeated two times before bone union was achieved during the third attempt. For each procedure, the IM tissue was sampled for histology as part of an ongoing prospective clinical study designed to explain IMT failures. Hence, a systematic histological examination of the tissue revealed major differences in the IM organization allowing to link IM alteration with IMT failure.

## 2. Case Report

A 40-year-old male with a history of lower limb discrepancy was referred for the treatment of a segmental bone defect of the left femur complicating a lengthening procedure. He had no comorbidities. A 9 cm long bone gap had been created by excessive rapid lengthening related to a technical malfunction of the elongation nail. A previous lengthening of the right femur had been achieved successfully several years before using the same technique. Since the lower-limb lengths were equal, an IMT was decided for bone reconstruction. There was no history of infection.

**Figure 1:**
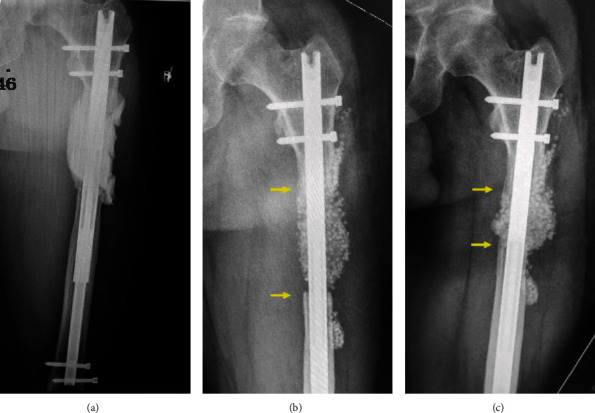
First procedure. Radiographs of the left femur showing the cement spacer (a), the graft aspect at five months after the first grafting (b), and the graft collapse following nail dynamization (c).

The first procedure (P1) was initiated at another institution with implantation of a cement spacer around the elongation nail (Figure 1(a)). Because of personal and medicolegal issues, the patient was referred 10 months later for bone grafting. Cancellous bone was harvested in the right femur using the Reamer/Irrigator/Aspirator (RIA) system (Synthes, West Chester, PA, USA) and mixed with 30 percent of synthetic bone substitute (TCH®; Kasios, L'Union, France). After five months, X-rays seemed to show a satisfying graft integration except at the distal edge (Figure 1(b)). Nail dynamization was performed by way of distal locking screw removal, and progressive weight-bearing was initiated. Two weeks later, the patient presented with pain and a 5 cm shortening of the left lower limb. X-rays revealed a major collapse of the graft (Figure 1(c)). A second IMT was decided.

**Figure 2:**
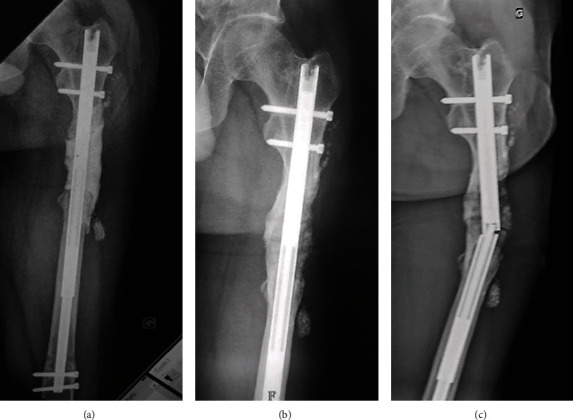
Second procedure. Radiographs showing the second cement spacer (a), the bone graft aspect at six months after the second grafting (b), and the subsequent elongation nail breakage that occurred four months later (c).

The first step of the second procedure (P2) consisted of complete removal of the soft and avascularized graft, restoration of femur length, repetition of distal nail locking, and implantation of a new cement spacer (Palacos®R+G; Heraeus, Hanau, Germany) into the defect (Figures 2(a) and 2(b)). A slight shortening was tolerated to limit the size of the defect. Eight weeks later, an iterative bone grafting was performed using a bilateral posterior iliac crest bone graft (ICBG) combined with allograft (Biobank, Presles-en-Brie, France). Progressive weight-bearing was allowed at the fourth month, full weight-bearing was allowed at the sixth month, and radiological bone union was considered acquired at the eighth month after the procedure, respectively. However, the patient continued to complain about pain until the breakage of the elongation nail occurred at 10 months after the second grafting (Figure 2(c)). A nail exchange with iterative IMT was selected.

**Figure 3:**
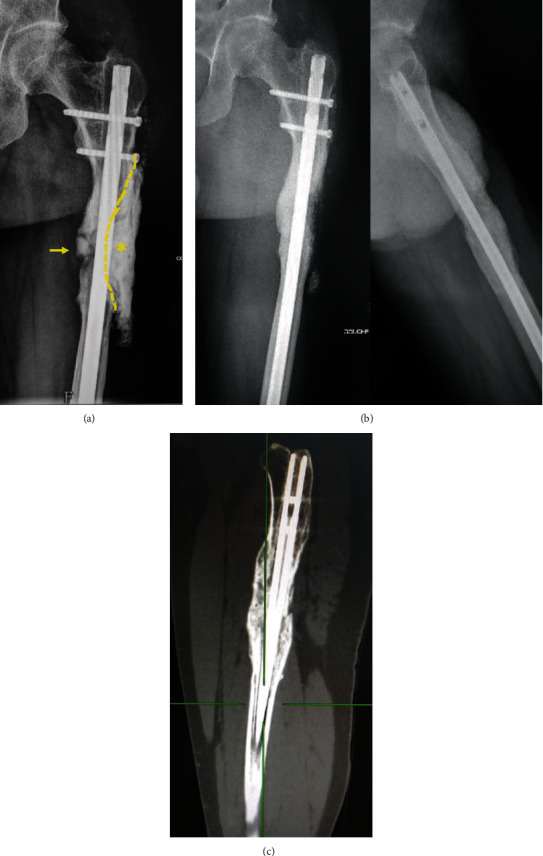
Third procedure. Radiograph showing a fracture of the integrated graft (arrow) and a partial cement spacer (star) into the residual lateral bone defect (a). X-rays (b) and CT-scan (c) demonstrating complete bone union at the last follow-up.

During the third procedure (P3), the graft was found to be lost on the anterolateral side of the femur. Conversely, the posteromedial side consisted of dense reconstructed bone with a clear fracture line. The broken nail was replaced by a monobloc conventional nail (T2 femoral nailing system, Stryker, Kalamazoo, MI, USA), and then, a third cement spacer (Palacos®R+G; Heraeus, Hanau, Germany) was prepared to fill in the lateral defect and wrap the bone extremities (Figure 3(a)). The last bone grafting was performed three months later using a bilateral anterior ICBG with allograft (Biobank, Presles-en-Brie, France). The same postoperative follow-up protocol was applied.

One year after the last procedure and four years following the first spacer implantation, X-rays and computerized tomography (CT) scan demonstrated a complete circumferential bone union with 1.5 cm shortening (Figures 3(b) and 3(c)). The patient walked with a crutch and corrective insole. He has complained about muscle pain and weakness related to a significant degree of quadriceps atrophy. His short musculoskeletal function assessment score was 81 [8].

**Figure 4:**
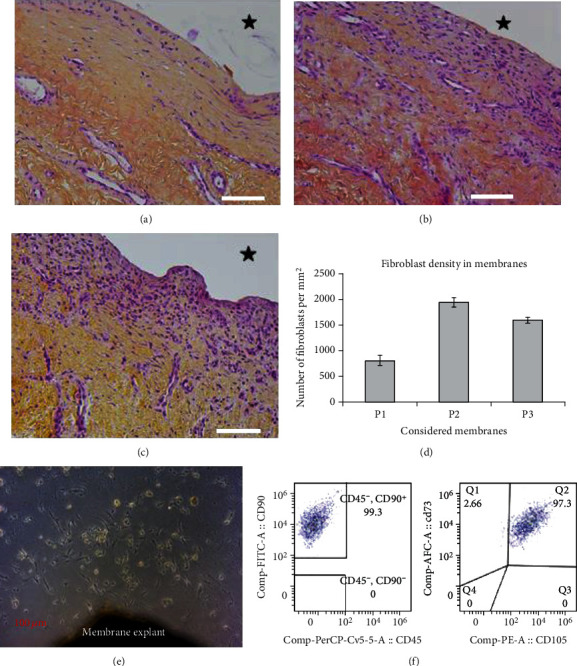
Histological sections of induced membranes from P1 (a), P2 (b), and P3 (c). After 4% paraformaldehyde fixation, membrane fragments were embedded in paraffin and then sections were cut and processed for routine hematoxylin-eosin-saffron staining. Magnification 20x. Scale bar = 100 *μ*m. The black star indicates the poly-methyl-methacrylate location before its removal. Fibroblast density is shown in (d) (mean number of fibroblasts per mm^2^ ± standard error of the mean from 3 different slides for each considered membrane). Plastic-adherent mesenchymal stromal cells were isolated according to the explant culture method (e); P2 membrane) and then characterized by flow cytometry according to their phenotypic profile: CD90^+^, CD73^+^, CD105^+^, and CD45^−^ (f).

The complications encountered in this case were not related to infection since systematic deep bacteriological samples taken during each step of each procedure were always sterile. In contrast, alterations in P1 membrane histology and cytometry were found. P2 and P3 membranes displayed typical IM morphologies as they were organized in two layers (Figure 4). The superficial layer in contact with PMMA contained many fibroblasts (1945 ± 95 and 1603 ± 53 cells per mm^2^ in P2 and P3, respectively) and a very few infiltrated macrophages (less than 10 cells per mm^2^). The deeper layer was composed of collagen embedding some fibroblasts and large blood vessels. Conversely, the superficial layer in the P1 membrane was strikingly different since fibroblast density was only 820 ± 94 cells per mm^2^ (Figure 4(d)). This layer was also thinner than the ones observed in P2 and P3 membranes. Furthermore, as assessed by cell culture explant and further flow cytometry analysis (Figures 4(e) and 4(f)), P2 and P3 membranes contained numerous mesenchymal stromal cells (MSCs, CD45^−^/CD90^+^/CD73^+^/CD105^+^ phenotype) while P1 membrane was completely deprived of these cells. The presence of MSCs is of importance since these cells give rise to osteoprogenitors and further mature osteoblasts responsible for bone formation after subsequent cell differentiation steps. Therefore, the absence of MSC in the P1 membrane may reflect an alteration in the osteogenic properties of this membrane.

## 3. Discussion

The special interest in this current case was the repetition of two consecutive IMT revisions to solve two technical imperfections that occurred in sequence in an unusual clinical situation. We acknowledge that the choice to perform the same method twice may be questionable, but we felt that alternative procedures were not suitable: a bone transport complication was the origin of the defect, and a vascularized fibular transfer was not compatible with the intramedullary fixation which has proved to be the best option for femur reconstruction [9]. In addition, we believe that the vascularized fibula should be used as a last resort in the lower limb (e.g., when no other autologous bone graft is available) since it is subject to specific complications including thrombosis of the anastomosed vessels, stress fracture, and delayed hypertrophy [10].

The main advantages of IMT include its simplicity, as it does not require any sophisticated equipment or microsurgical skills to perform, and a healing time independent of the defect length. However, according to Morelli et al. [6], IMT exhibits a failure rate of 10.3% and a complications rate of 49.6%, with cases mostly composed of persistent infections. They stressed the frequent need for reintervention, including additional union surgeries, which results in a variable healing time [6]. In fact, IMT responds to rigorous technical details involving each phase of the procedure [2, 4, 7]. In this case study, we demonstrated by means of clinical and histological observations that the first and the second IMT failures had two distinct origins, namely, biological and mechanical causes, respectively.

Our data show that the failure of P1 had a biological origin. Indeed, unlike P2 and P3 membranes, the P1 membrane has a strongly reduced number of fibroblasts and is deprived of MSCs. Since these two cell types are responsible for the secretion of growth factors involved in bone repair, their decreased numbers in the P1 membrane could be related to a diminution of secreted growth factors and then a subsequent detrimental bone repair [3, 11]. Moreover, the graft consistency during the first revision clearly indicated that its revascularization by the membrane had failed. The P1 IM failure can be explained by the extended 10-month induction period between the two stages, as the recommended interval ranges from four to six weeks [3, 12]. Recent studies found that IM biological properties decrease markedly with time and confirmed that bone grafting should not be delayed beyond the recommended period [2, 11, 13, 14]. On the other hand, successful results have been occasionally reported with late grafting [13]. Another explanation could be a defective immune response related to anti-inflammatory drug medication in the weeks following the too fast and painful limb lengthening. Furthermore, we did not use recombinant growth factors since mixing such adjuvants with the material graft could have a deleterious effect. We believe that localized high density of these products and possible effects of competition with secreted growth factors can lead to partial graft resorption [2, 15].

In contrast, the P2 failure was thought as likely to be of mechanical origin. Excessive mechanical constraints related to inadequate stability of the elongation nail have resulted in a failed integration on the lateral part of the graft during P2. Micromotions between the inner and outer tubes produced lateral bending stresses that precluded bone graft integration on the lateral cortex only. Conversely, bone regeneration was satisfying on the medial side of the graft not subjected to bending stresses. This elongation nail, which was not robust enough for bone defect reconstruction, should have been changed since the IMT was decided. A very stable degree of fixation is indeed required to promote membrane induction and graft revascularization [2, 12, 15]. Clinical observations and the assumption of mechanical failure were supported by the P2 membrane histological examination. Both the P2 and P3 membranes display similar histologies and comparable osteoprogenitor content, and this was related to satisfying the bone graft healing for both procedures.

To conclude, the IMT is simple to perform but its completion must be technically rigorous to avoid treatment failure. This unique case provides the evidence of a relationship between IM alteration and therapeutic failure and confirms that a stable fixation is critical to graft integration.

## References

[1] Masquelet A. C., Fitoussi F., Bégué T., Muller G. P. (2000). Reconstruction of the long bones by the induced membrane and spongy autograft. *Annales de Chirurgie Plastique et Esthétique*.

[2] Masquelet A. C. (2017). Induced membrane technique: pearls and pitfalls. *Journal of Orthopaedic Trauma*.

[3] Pelissier P., Masquelet A. C., Bareille R., Mathoulin-Pelissier S., Amedee J. (2004). Induced membranes secrete growth factors including vascular and osteoinductive factors and could stimulate bone regeneration. *Journal of Orthopaedic Research*.

[4] Giannoudis P. V., Faour O., Goff T., Kanakaris N., Dimitriou R. (2011). Masquelet technique for the treatment of bone defects: tips-tricks and future directions. *Injury*.

[5] Morris R., Hossain M., Evans A., Pallister I. (2017). Induced membrane technique for treating tibial defects gives mixed results. *The Bone & Joint Journal*.

[6] Morelli I., Drago L., George D. A., Gallazzi E., Scarponi S., Romanò C. L. (2016). Masquelet technique: myth or reality? A systematic review and meta-analysis. *Injury*.

[7] Mi M., Papakostidis C., Wu X., Giannoudis P. V. (2020). Mixed results with the Masquelet technique: a fact or a myth?. *Injury*.

[8] Swiontkowski M. F., Engelberg R., Martin D. P., Agel J. (1999). Short musculoskeletal function assessment questionnaire. *The Journal of Bone and Joint Surgery. American Volume*.

[9] Morwood M. P., Streufert B. D., Bauer A. (2019). Intramedullary nails yield superior results compared with plate fixation when using the Masquelet technique in the femur and tibia. *Journal of Orthopaedic Trauma*.

[10] Beris A. E., Lykissas M. G., Korompilias A. V. (2011). Vascularized fibula transfer for lower limb reconstruction. *Microsurgery*.

[11] Gouron R., Petit L., Boudot C. (2017). Osteoclasts and their precursors are present in the induced membrane during bone reconstruction using the Masquelet technique. *Journal of Tissue Engineering and Regenerative Medicine*.

[12] Masquelet A. C., Kishi T., Benko P. E. (2019). Very long-term results of post-traumatic bone defect reconstruction by the induced membrane technique. *Orthopaedics & Traumatology, Surgery & Research*.

[13] Aho O. M., Lehenkari P., Ristiniemi J., Lehtonen S., Risteli J., Leskelä H. V. (2013). The mechanism of action of induced membranes in bone repair. *The Journal of Bone and Joint Surgery. American Volume*.

[14] Assal M., Stern R. (2014). The Masquelet procedure gone awry. *Orthopedics*.

[15] Masquelet A. C., Bégué T. (2010). The concept of induced membrane for reconstruction of long bone defects. *The Orthopedic Clinics of North America*.

